# Serum Phosphate as a Risk Factor for Cardiovascular Events in People with and without Chronic Kidney Disease: A Large Community Based Cohort Study

**DOI:** 10.1371/journal.pone.0074996

**Published:** 2013-09-10

**Authors:** Andrew Peter McGovern, Simon de Lusignan, Jeremy van Vlymen, Harshana Liyanage, Charles Richard Tomson, Hugh Gallagher, Meena Rafiq, Simon Jones

**Affiliations:** 1 Department of Health Care Management and Policy, University of Surrey, Guildford, United Kingdom; 2 Division of Population Health Sciences and Education, St. George's – University of London, London, United Kingdom; 3 Department of Renal Medicine, Southmead Hospital – North Bristol NHS Trust, Bristol, United Kingdom; 4 South West Thames Renal and Transplantation Unit, St. Helier Hospital, Carshalton, United Kingdom; Innsbruck Medical University, Austria

## Abstract

**Background:**

Serum phosphate is a known risk factor for cardiovascular events and mortality in people with chronic kidney disease (CKD), however data on the association of these outcomes with serum phosphate in the general population are scarce. We investigate this relationship in people with and without CKD in a large community-based population.

**Methods:**

Three groups from an adult cohort of the Quality Improvement in Chronic Kidney Disease (QICKD) cluster randomised trial (ISRCTN56023731) were followed over a period of 2.5 years: people with normal renal function (N = 24,184), people with CKD stages 1–2 (N = 20,356), and people with CKD stages 3–5 (N = 13,292). We used a multilevel logistic regression model to determine the association between serum phosphate, in these groups, and a composite outcome of all-cause mortality, cardiovascular events, and advanced coronary artery disease. We adjusted for known cardiovascular risk factors.

**Findings:**

Higher phosphate levels were found to correlate with increased cardiovascular risk. In people with normal renal function and CKD stages 1–2, Phosphate levels between 1.25 and 1.50 mmol/l were associated with increased cardiovascular events; odds ratio (OR) 1.36 (95% CI 1.06–1.74; p = 0.016) in people with normal renal function and OR 1.40 (95% CI 1.09–1.81; p = 0.010) in people with CKD stages 1–2. Hypophosphatemia (<0.75 mmol/l) was associated with fewer cardiovascular events in people with normal renal function; OR 0.59 (95% CI 0.36–0.97; p = 0.049). In people with CKD stages 3–5, hyperphosphatemia (>1.50 mmol/l) was associated with increased cardiovascular risk; OR 2.34 (95% CI 1.64–3.32; p<0.001). Other phosphate ranges were not found to have a significant impact on cardiovascular events in people with CKD stages 3–5.

**Conclusions:**

Serum phosphate is associated with cardiovascular events in people with and without CKD. Further research is required to determine the mechanisms underlying these associations.

## Introduction

Observational data suggest that an elevated serum phosphate increases the risk of cardiovascular events and mortality in patients with chronic kidney disease (CKD) [1]–[4]. Furthermore, hypophosphatemia is associated with reduced cardiovascular events in people with CKD [1], [3]. Hyperphosphataemia is also associated with increased vascular and valvular calcification in end-stage renal disease [5], [6]. Secondary hyperparathyroidism is common in people with CKD and subsequently altered calcium and vitamin D metabolism may be responsible for this increased arterial calcification [7]. However, repeated investigation has not been able to identify a relationship between serum calcium and cardiovascular events, and there is inconclusive evidence for an association with parathyroid hormone levels [3].

Given the diverse biochemical roles of phosphate, it is possible that high serum phosphate is more directly responsible for cardiovascular events than previously suggested. Indeed, elevated phosphate also correlates with increased vascular and valvular calcification in people with normal renal function [8], [9] and an association with cardiovascular events has also been reported in people with pre-existing coronary artery disease [10] and in the general population [11]–[13].

A number of potential mechanisms by which phosphate leads to increased cardiovascular risk have been proposed [14]. Elevated phosphate levels induce degradation of the extracellular matrix and causes osteochondrogenic change in vascular smooth muscle cells [15]. These changes cause increased deposition of extracellular calcium phosphate crystals, cell apoptosis, and ultimately vascular calcification [15], [16]. Vessel calcification is associated with left ventricular hypertrophy, decreased coronary blood flow, and cardiovascular events although causality has not been determined [17], [18]. Hyperphosphatemia may also cause endothelial damage through increased production of reactive oxygen species [19], [20]. This process is potentially preventable as dietary phosphate restriction in murine models of secondary hyperparathyroidism prevents endothelial damage [21], [22]. Population studies demonstrate that increased dietary phosphate intake is correlated with increased serum phosphate; particularly phosphate additives in processed foods [23], [24].

The major focus of observational studies has so far been on patients with CKD, however a number of studies have analysed the correlation with phosphate and cardiovascular events and mortality in the general population. In order to confirm or refute previous findings, we investigate the impact of phosphate in the general population with a large community based cohort, using routinely collected data. We also compare these results with the relationship between phosphate and cardiovascular events in people with CKD. As parathyroid hormone (PTH) is involved in phosphate homeostasis we also investigate the relationship between serum PTH and cardiovascular events.

## Methods

The data analysed were from the Quality Improvement in Chronic Kidney Disease (QICKD) cluster randomised trial (clinical trials registration: ISRCTN56023731) [25]. These data consist of the primary care records of the population of 127 primary care practices across England; a nationally representative sample of urban, sub-urban and rural practices in London, Surrey, Sussex, Leicester, Birmingham and Cambridge. The complete protocol used for sampling and data collection from these practices for the QICKD trial have been previously described [26]. A sub-population of individuals in whom phosphate and renal indices were available was selected for inclusion in this study. Routine data from electronic records were collected over a five year period; between January 2006 and December 2010. Data recorded between January 2006 and June 2008 was used to determine the baseline characteristics of the people included in the study, with the most recent values of creatinine, phosphate and PTH used for comparison. A second data collection was undertaken at 30 months to obtain follow-up data on outcomes.

The study population was subdivided by renal function into three groups; people with normal renal function, people with CKD stage 1–2, and people with CKD stage 3–5. CKD stage was identified using estimated glomerular filtration rate (eGFR) measurements calculated using the modified diet in renal disease equation from serum creatinine measurements [27], [28] and measurements of proteinuria. Normal renal function was defined as an eGFR ≥90 ml/min and absence of significant proteinuria. CKD stage 1–2 was defined as an eGFR of 60–89 ml/min or the presence of significant proteinuria with an eGFR ≥90 ml/min. CKD stages 3–5 were defined as an eGFR<60 ml/min. Proteinuria was analysed using the diagnostic criteria described by the National Institute for Health and Clinical Excellence with lower significance thresholds in people with diabetes (NICE) [28]. People without creatinine measurements were excluded from the analysis. People were only included in the normal renal function group if no proteinuria was present; those with no proteinuria measurements were excluded from this group.

### Outcomes

We used a combined outcome measure of all-cause mortality and incident stroke, transient ischaemic attack (TIA), myocardial infarction (MI), advanced coronary artery disease, new cardiac failure and death, during the follow up period. Advanced coronary artery disease was defined as at least one of; coronary artery revascularisation procedures, progressive angina, angina at rest, and acute coronary syndrome not otherwise diagnosed as MI.

### Predictors

Traditional cardiovascular risk factors were identified from analysis of the electronic patient records at baseline. These were the Framingham risk factors; age, ethnicity, smoking status, alcohol use, and body mass index (BMI), diabetes, hypertension, low density lipoprotein (LDL) cholesterol, high density lipoprotein (HDL) cholesterol, and total cholesterol (TC).

BMI was calculated from routinely gathered measurements of weight and height performed by physicians and practice nurses. The presence of diabetes was defined from clinical coding records and serum glucose values using a method we have validated previously [29]. The presence of significant hypertension was defined by prescription of antihypertensive medications (angiotensin converting enzyme inhibitors and angiotensin II receptor blockers) using electronic prescription records kept by every practice included in the study.

Serum phosphate levels were analysed as a categorical variable grouped in 0.25 mmol/l bands (<0.75, 0.75–1.00, 1.01–1.25, 1.26–1.50, and >1.50 mmol/l). Serum PTH was categorised as high (>60 ng/l), normal (10–60 ng/l), low (<10 ng/l) or not measured.

The presence of proteinuria was determined by examining a hierarchy of clinical tests; albumin creatinine ratio (ACR), then if not available; protien creatinine ratio (PCR), 24 hour urinary protein, or urine dipstick testing in turn. Threshold values are shown in Table 1. The lower significance thresholds for people with diabetes are also shown [28].

**Table 1:** Protienuria threshold values by clinical test. Threshold values are adapted from the 2008 NICE guidelines [28] and Lamb et al. [45].

Clinical test	Non diabetes threshold value	Diabetes threshold value
Albumin creatinin ratio (mg/mmol)	≥30	≥2.5 males, ≥3.5 females
Protein creatinine ratio (mg/mmol)	≥50	≥15
24 h urinary protein (mg/24h)	≥300	≥150
Urine dipstick testing	‘trace’ or above	‘trace’ or above

Laboratory sample data (cholesterol, glucose, phosphate, creatinine, and proteinuria measurements) were taken from samples collected routinely in primary care practices during practice opening times (usually 8am to 6pm) and processed in local laboratories. Electronic primary care records are automatically updated with these results. Where multiple values of each parameter were recorded during the baseline period the most recent value was used.

### Statistical analysis

Numerical data was refined, to adjust for inputting errors, by removing values outside of physiological limits. Where the variable of interest had not been measured or recorded in the patient record individuals were categorised as being not monitored for that given parameter and included separately for analysis.

Multilevel binary logistic regression models were built to account for outcome variation between primary care practices. Patients were nested within practices using a random intercept. Model selection was performed using the approach described by Maindonald and Braun [30] by minimising the Bayesian information criterion (BIC) using backward stepwise elimination. Models were validated using receiver operating characteristic (ROC) curves and Hosmer-Lemeshow testing. People who left their practice during the follow up period were excluded from the logistic regression analysis. The analysis was performed using the statistical package R and the multilevel R package lme4 [31].

Cox proportional hazards multivariate models were also constructed using the statistical package R to provide a time-event analysis. Individuals who left their practice during the follow up period were included as censored data. The date of each outcome event was taken as the date it was recorded as occurring in the primary care records. This data was available for all outcome events.

### Ethical considerations

No patient identifiable data was used in the analysis described here. Other ethical considerations of the QICKD study are described elsewhere [26], [32].

## Results

A total of 741,913 people were included in the analysis. We excluded people who died or left the practice before the start of the follow up period (n = 123), if they were aged less than 18 at the start of the follow up period (n = 142,533), or those who left the practice during the follow up period (n = 51,763). From the remaining adults (N = 547,494) only people with phosphate measurements and required renal indices measurements before the start of the follow up period were included (N = 57,832). The proportion of the complete population with measurements of each variable is given in table 2. From these; 24,184 people were found to have no evidence of renal impairment, 20,356 people CKD stages 1–2, and 13,292 people CKD stages 3–5. The demographics of these groups are shown in table 2.

**Table 2:** Demographics of people included for analysis in each study group; those with normal renal function (N = 24,184), those with CKD stages 1–2 (N = 20,356), and those with CKD stages 3–5 (N = 13,292) where phosphate measurements were available.

	Total population	Normal renal function	CKD stages 1–2	CKD stages 3–5
	n (%)	n (%)	n (%)	n (%)
Female	271,503 (49.6)	14,082 (58.2)	12,330 (60.6)	9,057 (68.1)
Age: mean (SD)	49.0 years (±17.8)	52.8 years (±17.0)	56.0 years (±16.2)	72.8 years (±12.9)
Never smoked	181,662 (33.2)	9,552 (39.5)	9,266 (45.5)	5,950 (44.8)
Current Smoker	94,042 (17.2)	5,074 (21.0)	3,813 (18.7)	1,720 (12.9)
Ex-smoker	49,584 (9.1)	2,833 (11.7)	2,643 (13.0)	2,539 (19.1)
Diabetes	33,811 (6.2)	1,953 (8.1)	3,163 (15.5)	2,536 (19.1)
Antihypertensive medication	77,056 (14.1)	4,503 (18.6)	5,354 (26.3)	7,388 (55.6)
HDL cholesterol: measured	167,607 (30.6)	14,502 (60.0)	14,281 (70.2)	10,321 (77.6)
HDL cholesterol: mean (SD)	1.44 mmol/l (±0.43)	1.45 mmol/l (±0.42)	1.41 mmol/l (±0.41)	1.46 mmol/l (±0.43)
Creatinine: measured	234,800 (42.9)	24,184 (100.0)	20,356 (100.0)	13,292 (100.0)
Protienuria: measured	231,925 (42.3)	24,184 (100.0)	14,290 (70.2)	8,995 (67.7)
Phosphate: measured	57,832 (10.6)	24,184 (100.0)	20,356 (100.0)	13,292 (100.0)
Phosphate: <0.75 mmol/l	1,340 (0.2)	627 (2.6)	522 (2.6)	273 (2.1)
Phosphate: 0.75–1.00 mmol/l	14,601 (2.7)	6,726 (27.8)	5,506 (27.0)	3,270 (24.6)
Phosphate: 1.00–1.25 mmol/l	27,562 (5.0)	12,201 (50.5)	10,405 (51.1)	6,750 (50.8)
Phosphate: 1.25–1.50 mmol/l	9,943 (1.8)	4,295 (17.8)	3,644 (17.9)	2,687 (20.2)
Phosphate: >1.50 mmol/l	861 (0.2)	335 (1.4)	279 (1.4)	312 (2.3)
PTH measured	1,633 (0.3)	225 (0.9)	250 (1.2)	764 (5.7)

For comparison, the demographics of the total adult population (N = 547,494) from which these groups are extracted is also included. SD  =  standard deviation.

The mean age of the group with normal renal function was 52.8 years (standard deviation 17.0 years). 1,005 (4.2%) people suffered one or more adverse cardiovascular event during the 30 month follow up period. There were 133 strokes, 120 TIAs, 84 MIs, 110 coronary artery procedures, 45 other advanced coronary artery disease events, 77 new cases of heart failure and 521 deaths.

The mean age of the population with CKD stage 1–2 was 56.0 years (standard deviation 16.1 years). 861 (4.2%) people with CKD suffered one or more adverse cardiovascular event including 116 strokes, 114 TIAs, 79 MIs, 109 coronary artery procedures, 27 other advanced coronary artery disease events, 79 new cases of heart failure and 415 deaths.

The mean age of the population with CKD stage 3–5 was 72.8 years (standard deviation 12.9 years). 1,600 (12.0%) people with CKD suffered one or more adverse cardiovascular event including 175 strokes, 140 TIAs, 120 MIs, 113 coronary artery procedures, 50 other advanced coronary artery disease events, 143 new cases of heart failure and 986 deaths. The total number of outcome events by CKD stage is given in table 3).

**Table 3:** Number of outcome events in each study group by phosphate range in; the total population (N = 57,832), those with normal renal function (N = 24,184), those with CKD stages 1–2 (N = 20,356), and those with CKD stages 3–5 (N = 13,292) where phosphate measurements were available.

	Total population	Normal renal function	CKD stages 1–2	CKD stages 3–5
	n (%)	n (%)	n (%)	n (%)
Phosphate: <0.75 mmol/l	68 (4.8)	15 (2.4)	19 (3.6)	34 (12.5)
Phosphate: 0.75–1.00 mmol/l	872 (5.6)	264 (3.9)	223 (4.1)	385 (11.8)
Phosphate: 1.00–1.25 mmol/l	1,721 (5.9)	517 (4.2)	435 (4.2)	769 (11.4)
Phosphate: 1.25–1.50 mmol/l	706 (6.6)	192 (4.5)	172 (4.7)	342 (12.7)
Phosphate: >1.50 mmol/l	99 (10.7)	17 (5.1)	12 (4.3)	70 (22.4)

The percentages shown are the proportion of people within category who had a cardiovascular event (not of the group population).

Serum PTH levels, BMI and total cholesterol were not found to be significantly associated with adverse outcomes and were therefore removed from the regression models. No variables were found to be co-linear with phosphate.

**Figure 1:**
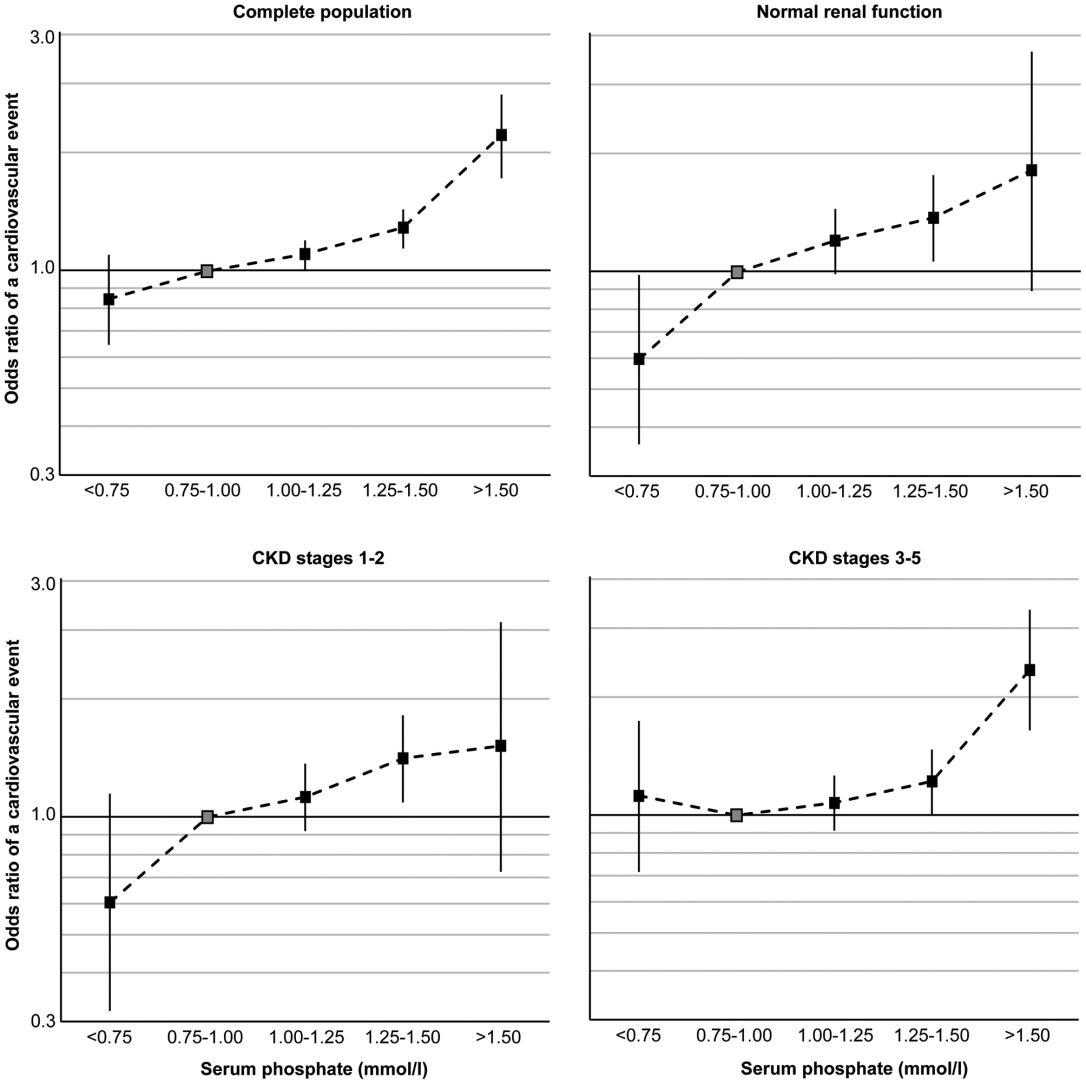
The adjusted odd ratio of a cardiovascular event during a 30 month follow up period for the complete population and sub populations; 24,184 people with normal renal function, 20,356 people with CKD stages 1–2, and 13,292 people with CKD stages 3–5 by serum phosphate category.

After adjusting for known confounders, both logistic regression models (table 4) and Cox regression models (table 5) demonstrate consistent correlations between phosphate and cardiovascular risk in all three population groups: A monotonically increasing relationship between phosphate and cardiovascular events was found in people with normal renal function (figure 1). Lower phosphate appears to be protective and higher phosphate increases risk in this group. This trend also appears amongst people with CKD stage 1–2, however reduced risk with lower phosphate levels was not statistically significant. In people with CKD stages 3–5 hyperphosphatemia (which we have defined as phosphate >1.5 mmol/l) was found to be associated with increased cardiovascular risk. No protective effect of low phosphate (which we have defined as <0.75 mmol/l) is suggested in people with CKD stages 3–5 (figure 1).

**Table 4:** Logistic regression analysis: The clinical characteristics of people with normal renal function, CKD stages 1–2, and people with CKD stages 3–5 and odds ratio of cardiovascular events and mortality during 30 months of follow up.

**Model performance:**	**Complete population**		**Normal renal function**		**CKD stages 1–2**		**CKD stages 3–5**	
Bayesian information criteria	21,510		4,991		4,803		7,090	
-log-likelihood	10,673		2,428		2,335		3,480	
ROC curve statistic	0.833		0.822		0.811		0.7465	
**Random effects:**								
Random intercepts for primary care practice:								
Variance	0.199		0.165		0.160		0.179	
Standard deviation	0.446		0.406		0.400		0.423	
**Fixed effects:**	**Odds Ratio (Confidence interval)**	**P value**	**Odds Ratio (Confidence interval)**	**P value**	**Odds Ratio (Confidence interval)**	**P value**	**Odds Ratio (Confidence interval)**	**P value**
Female	1.00 [reference]		1.00 [reference]		1.00 [reference]		1.00 [reference]	
Male	1.70 (1.57–1.84)	<0.001	2.01 (1.68–2.40)	<0.001	1.76 (1.47–2.11)	<0.001	1.44 (1.25–1.66)	<0.001
Age (years)	1.08 (1.08–1.09)	<0.001	1.08 (1.07–1.09)	<0.001	1.08 (1.07–1.09)	<0.001	1.07 (1.06–1.08)	<0.001
Never smoked	1.00 [reference]		1.00 [reference]		1.00 [reference]		1.00 [reference]	
Current Smoker	1.60 (1.44–1.78)	<0.001	1.49 (1.21–1.84)	<0.001	1.62 (1.30–2.02)	<0.001	1.46 (1.20–1.77)	<0.001
Ex-smoker	1.19 (1.08–1.32)	<0.001	0.97 (0.77–1.22)	0.775	1.21 (0.97–1.52)	0.091	1.32 (1.12–1.55)	<0.001
Diabetes	1.43 (1.30–1.57)	<0.001	1.43 (1.16–1.76)	<0.001	1.27 (1.05–1.54)	0.014	1.44 (1.24–1.66)	<0.001
Antihypertensive medication	1.25 (1.16–1.36)	<0.001	1.20 (1.01–1.43)	0.036	1.32 (1.11–1.56)	0.001	1.16 (1.01–1.33)	0.040
HDL Cholesterol	1.25 (1.16–1.36)	<0.001	0.73 (0.60–0.90)	0.004	0.87 (0.70–1.08)	0.203	0.77 (0.65–0.91)	0.002
Phosphate: <0.75 mmol/l	0.83 (0.64–1.09)	0.175	0.59 (0.36–0.97)	0.049	0.60 (0.32–1.14)	0.117	1.11 (0.71–1.73)	0.647
Phosphate: 0.75–1.00 mmol/l	1.00 [reference]		1.00 [reference]		1.00 [reference]		1.00 [reference]	
Phosphate: 1.00–1.25 mmol/l	1.09 (0.99–1.19)	0.069	1.19 (0.98–1.43)	0.077	1.12 (0.92–1.36)	0.270	1.07 (0.91–1.25)	0.420
Phosphate: 1.25–1.50 mmol/l	1.27 (1.13–1.42)	<0.001	1.36 (1.06–1.74)	0.016	1.40 (1.09–1.81)	0.010	1.21 (1.00–1.46)	0.054
Phosphate: >1.50 mmol/l	2.19 (1.72–2.80)	<0.001	1.80 (0.89–3.63)	0.100	1.51 (0.72–3.13)	0.272	2.34 (1.64–3.32)	<0.001

**Table 5:** Cox regression analysis: The clinical characteristics of people with normal renal function and people with CKD stages 3–5, CKD stages 1–2, and odds ratio of cardiovascular events and mortality during 30 months of follow up.

Clinical characteristic	Complete population		Normal renal function		CKD stages 1–2		CKD stages 3–5	
	Hazard Ratio (Confidence interval)	P value	Hazard Ratio (Confidence interval)	P value	Hazard Ratio (Confidence interval)	P value	Hazard Ratio (Confidence interval)	P value
Female	1.00 [reference]		1.00 [reference]		1.00 [reference]		1.00 [reference]	
Male	1.58 (1.45–1.72)	<0.001	1.08 (1.07–1.08)	<0.001	1.08 (1.07–1.08)	<0.001	1.06 (1.05–1.07)	<0.001
Age (years)	1.07 (1.07–1.08)	<0.001	1.89 (1.60–2.24)	<0.001	1.66 (1.40–1.97)	<0.001	1.43 (1.26–1.61)	<0.001
Never smoked	1.00 [reference]		1.00 [reference]		1.00 [reference]		1.00 [reference]	
Current Smoker	1.50 (1.35–1.66)	<0.001	1.43 (1.18–1.73)	<0.001	1.62 (1.32–1.98)	<0.001	1.43 (1.21–1.68)	<0.001
Ex-smoker	1.22 (1.10–1.35)	<0.001	0.99 (0.80–1.22)	0.909	1.22 (0.99–1.49)	0.059	1.31 (1.14–1.50)	<0.001
Diabetes	1.32 (1.20–1.44)	<0.001	1.39 (1.15–1.68)	<0.001	1.20 (1.00–1.43)	0.048	1.37 (1.21–1.55)	<0.001
Antihypertensive medication	1.23 (1.13–1.33)	<0.001	1.14 (0.97–1.34)	0.108	1.26 (1.07–1.47)	0.004	1.16 (1.03–1.31)	0.017
HDL Cholesterol	0.78 (0.70–0.86)	<0.001	0.68 (0.56–0.83)	<0.001	0.87 (0.71–1.06)	0.175	0.80 (0.70–0.93)	0.003
Phosphate: <0.75 mmol/l	0.80 (0.60–1.07)	0.127	0.58 (0.36–0.97)	0.049	0.62 (0.34–1.14)	0.127	1.10 (0.74–1.63)	0.629
Phosphate: 0.75–1.00 mmol/l	1.00 [reference]		1.00 [reference]		1.00 [reference]		1.00 [reference]	
Phosphate: 1.00–1.25 mmol/l	1.11 (1.01–1.22)	0.031	1.19 (1.00–1.42)	0.054	1.09 (0.91–1.31)	0.341	1.08 (0.94–1.24)	0.303
Phosphate: 1.25–1.50 mmol/l	1.29 (1.15–1.45)	<0.001	1.38 (1.09–1.73)	0.007	1.31 (1.03–1.66)	0.026	1.24 (1.05–1.47)	0.014
Phosphate: >1.50 mmol/l	2.20 (1.75–2.78)	<0.001	1.62 (0.86–3.07)	0.138	1.44 (0.73–2.83)	0.288	2.40 (1.82–3.16)	<0.001

## Discussion

### Principal findings

In this large community-based population, serum phosphate levels were associated with cardiovascular events and mortality in both people with normal renal function and those with CKD. In people with normal renal function the relationship appears to be linear with no minimum threshold for improved cardiovascular outcomes. In those with CKD stages 3–5, hyperphosphatemia was associated with increased risk but lower levels of phosphate were not associated with reduced risk of cardiovascular events. In those with CKD stages 1–2 the relationship also appears to be a linear increase in cardiovascular risk with increasing phosphate although wide confidence intervals make this somewhat uncertain. This general trend between increased cardiovascular risk and higher serum phosphate remains when the population is analysed as a whole, but no reduction in risk was found with hypophosphatemia.

### Implications

These findings add to the growing weight of evidence that phosphate is an independent predictor of cardiovascular disease, in both people with and without CKD. Furthermore, these findings call in to question the definition of a ‘normal range’ for phosphate, as ‘low’ phosphate is associated with better outcomes in people with normal renal function. If phosphate is demonstrated to be an effective cardiovascular target, perception of serum levels should be similar to that of serum cholesterol or blood pressure, where the focus is on achieving values below a threshold. We propose that an increased research focus on the relationship between serum phosphate and cardiovascular events in the general population is needed.

### Comparison with the literature

We found the prevalence of CKD stages 3–5 to be 7.6% in the adult population; this is in good agreement with the UK based population estimate of 6%, which includes under 18 s [33]. Correlations with known cardiovascular risk factors were also consistent with those derived from the Framingham studies [34].

Previous data suggests increased phosphate is associated with increased mortality and cardiovascular risk in the general population and that hypophosphatemia is associated with reduced risk [10], [11]. This study confirms these findings. It also is in agreement with previous findings that hyperphosphatemia is associated with increased cardiovascular risk in people with CKD [1]–[3]. In people with CKD, phosphate below the normal range has been associated with both improved [1], [35] and worsened [36] cardiovascular outcomes. We found no effect of low phosphate on cardiovascular outcomes in the CKD population. Low phosphate is a marker of malnutrition [37], which is common in people with advanced renal disease [38]. This correlation may mask any potential benefits of low phosphate in this group and account for discrepancies between previous studies.

### Limitations of the method

The limitations of this study include those of working with routinely collected data (34). A particular consideration here is that only a small proportion of the population had serum phosphate measurements. There is a probable bias for increased co-morbidities amongst this group, however, this is likely to be a systematic effect and is therefore unlikely to explain the relationship between phosphate and cardiovascular events observed.

The number of people with phosphate levels at the extremes of the range was small. There is therefore greater uncertainty in our calculated odds of a cardiovascular event at these extremes. Additionally, very few people had serum PTH measurements. It is therefore not possible to conclude from these data that there is no relationship between serum PTH and cardiovascular events.

Serum phosphate levels are subject to diurnal variation with a minimum level in the morning with a subsequent rise throughout the day [39], [40]. As we did not have data on the times at which individual serum samples were taken, we were unable to adjust for this diurnal variation. It is likely that this will have weakened the association with cardiovascular outcomes. Fixed time sampling (e.g. 9am phosphate levels) may demonstrate improved correlation with cardiovascular outcomes. However, our data suggests that a random day-time phosphate measurement can be correlated with cardiovascular outcome.

### Further research

Whilst the mechanisms underlying this association remain to be fully elucidated the possibility that phosphate represents a potential therapeutic target should be considered. Trials looking at the impact of phosphate binders and dietary phosphate restriction have focused on end-stage renal disease and have mostly been underpowered for detection of reduced cardiovascular events [41]–[43], although sevelamer (a phosphate binder) has been demonstrated to reduce all-cause mortality in CKD stages 3–4 [44]. Research is needed into the causes of variation in phosphate levels within the population as this could prove to be vital in guiding potential intervention strategies. The role of phosphate additives in processed foods is one potential consideration.

## Conclusions

Serum phosphate is a predictor of cardiovascular events in people with and without CKD: High phosphate levels are associated with increased cardiovascular risk in both those with normal renal function and CKD. Low phosphate levels are associated with fewer cardiovascular events in people without renal disease. Further research is required to determine the mechanisms underlying these associations.
